# Salvage treatment of a metastatic colorectal cancer with pMMR/MSS in a 21-year-old man: a case report

**DOI:** 10.3389/fonc.2025.1567760

**Published:** 2025-08-22

**Authors:** Xuesong Li, Hao Liu, Zhuo Yu

**Affiliations:** ^1^ Department of Medical Oncology, Beijing Tsinghua Changgung Hospital, School of Clinical Medicine, Tsinghua Medicine, Tsinghua University, Beijing, China; ^2^ Department of Pathology, Beijing Tsinghua Changgung Hospital, School of Clinical Medicine, Tsinghua Medicine, Tsinghua University, Beijing, China

**Keywords:** colorectal cancer, proficient mismatch repair/microsatellite stability, PD-1 inhibitor, chidamide, treatment

## Abstract

In metastatic colorectal cancer (mCRC) patients with proficient mismatch repair (pMMR)/microsatellite stability (MSS), beyond third-line therapies were extremely limited. Here, we reported a case of a 21-year-old male patient with pMMR/MSS mCRC who failed to respond to both first- and second-line treatment and subsequently received non-standard third-line therapy at a local hospital. This patient was referred to our hospital, and we initiated salvage therapies. The fourth-line treatment, including tislelizumab, regorafenib, pemetrexed, and raltitrexed, was administered with a progression-free survival (PFS) of 13 months. Then, this patient received fifth-line treatment with chidamide, fruquintinib, toripalimab, raltitrexed, and nanoparticle albumin-bound paclitaxel with a PFS of 8 months. During the whole treatment, side effects were tolerable and significantly alleviated with appropriate symptomatic therapies. In addition, cystoscopy plus transurethral resection of a metastatic bladder tumor was successfully conducted to halt the bleeding. The sixth-line regimen was started, and he is still under follow-up. Collectively, this patient achieved long-term survival with a high quality of life through therapies beyond the third line.

## 1 Introduction

The latest studies have demonstrated that programmed cell death protein 1 (PD-1) antibodies have exhibited significant therapeutic benefits in metastatic colorectal cancer (mCRC) patients with mismatch repair deficiency (dMMR) or microsatellite instability-high (MSI-H) as first-line treatment (1). However, the majority of CRC patients show a microsatellite-stable (MSS) or proficient in mismatch repair (pMMR) status with a limited mutational burden and less tumor-infiltrating lymphocytes, resulting in a significantly constrained efficacy of immune checkpoint inhibitors (ICIs) (2). Currently, standard first- and second-line therapeutic approaches for mCRC patients involve oxaliplatin- and fluoropyrimidine-based chemotherapies, often combined with anti-epidermal growth factor receptor (EGFR) or anti-vascular endothelial growth factor (VEGF)-targeted therapies (3). For individuals with mCRC, the main aims of treatment are to extend overall survival (OS) and to elevate quality of life (QoL). Although there is agreement on the standard first- and second-line treatment for MSS mCRC among oncologists around the world, the options for third- or later-line treatment are extremely limited. Only regorafenib, fruquintinib, and trifluridine/tipiracil (FTD/TPI, TAS-102) are recommended by guidelines, and the median PFS (mPFS) is merely approximately 2–3 months (4–8). Here, we reported a 21-year-old male patient with mCRC who failed to respond to both first- and second-line treatment. However, the local hospital where the patient visited had limited medical care, and he received non-standard third-line treatment. We initiated treatments beyond the third line as salvage therapy settings.

## 2 Case report

**Figure 1:**
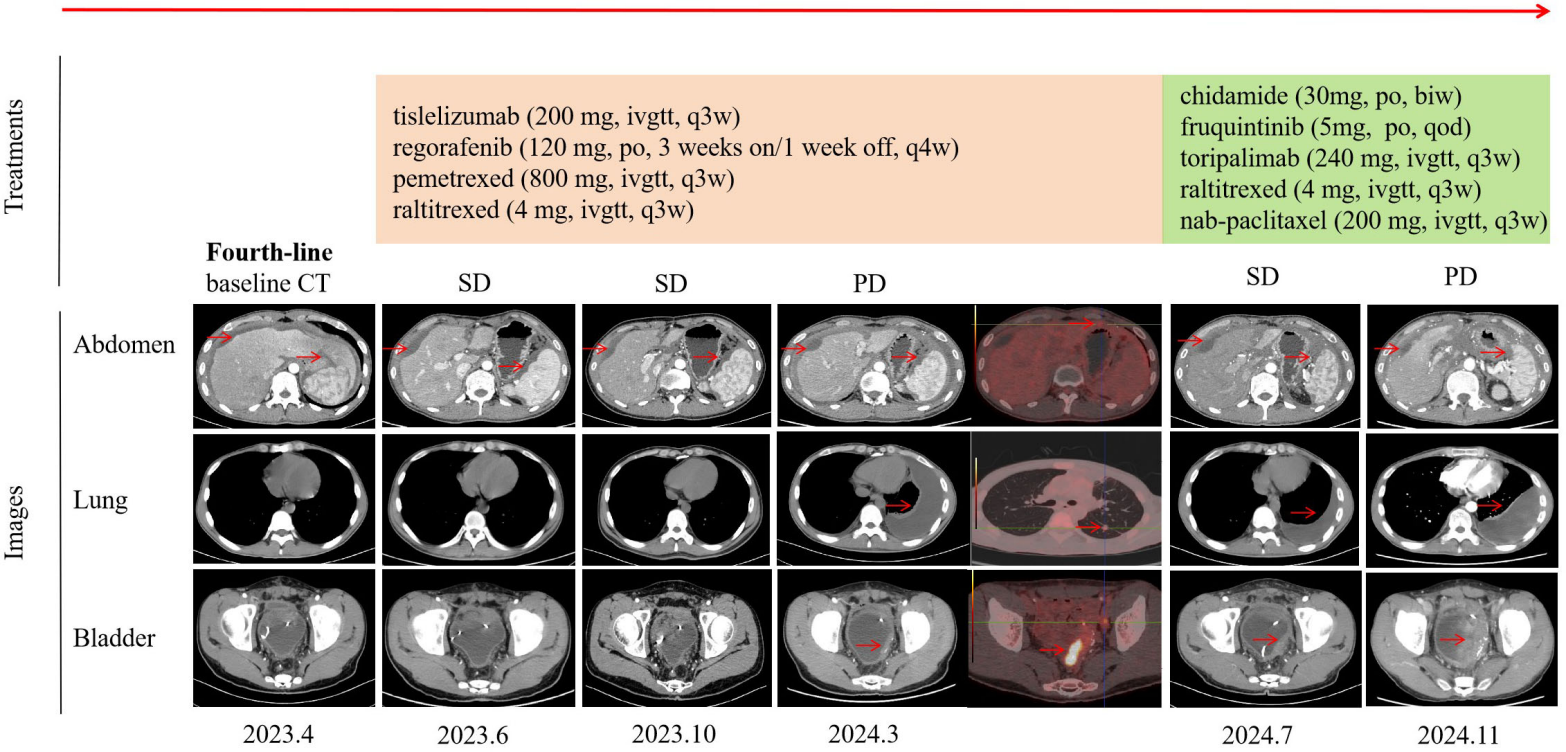
Timeline of treatment and efficacy evaluation. The “Treatments” section detailed the timeline of therapeutic regimens administered. The “Images” section included chest, abdomen, and pelvis CT scans, as well as positron emission tomography images at various stages of disease progression. Efficacy was evaluated according to the RECIST criteria. Tumor lesions are highlighted by red arrows. CT, computed tomography; SD, stable disease; PD, progression disease.

**Figure 2:**
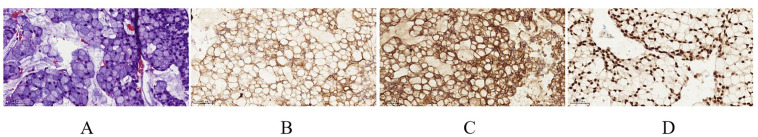
**(A)** Tumor tissue (hematoxylin and eosin staining, magnification ×400). **(B)** CK19 (magnification ×400). **(C)** CK20 (magnification ×400). **(D)** CDX-2 (magnification ×400).

A 21-year-old man was diagnosed with adenocarcinoma with a signet-ring cell component of the sigmoid colon (T4N0M1) in May 2022 at a local hospital, with metastasis to the peritoneum. Next-generation sequencing (NGS) showed *KRAS*, *NRAS*, *BRAF* wild type, and MSS. He initiated CapeOx chemotherapy (oxaliplatin and capecitabine) for three cycles and FOLFOX (5-fluorouracil, leucovorin, oxaliplatin) plus cetuximab for two cycles, after which he experienced a progressive disease. Then, second-line treatment (FOLFOX plus bevacizumab) was administered for three cycles. An abdomen-pelvis computed tomography (CT) scan revealed peritoneal effusion and local progression, which led to a progressive disease. This patient received a third-line regimen with FOLFIRI (5-fluorouracil, leucovorin, irinotecan) and bevacizumab for three cycles. A CT scan found a reduction in the thickness of the sigmoid colon wall. He subsequently underwent total colectomy with partial resection of the ileum and rectum. On 24 April 2023, this patient was referred to our hospital for further treatment. Fourth-line treatment, consisting of tislelizumab (200 mg every 3 weeks), regorafenib (120 mg, 3 weeks on/1 week off), pemetrexed (800 mg every 3 weeks), and raltitrexed (4 mg every 3 weeks), was started. After a total of three cycles, he achieved a stable disease. He continued with this regimen for 10 cycles until 15 March 2024, when pleural effusion and metastases of the lung and bladder were observed, with a progression-free survival (PFS) of 13 months. The side effect was only a mild skin rash. This patient received fifth-line treatment with chidamide (30 mg, twice a week), fruquintinib (5 mg, every other day), toripalimab (240 mg every 3 weeks), raltitrexed (4 mg every 3 weeks), and nanoparticle albumin-bound paclitaxel (nab-paclitaxel) (200 mg every 3 weeks) for six cycles with stable disease. The side effect was mild bone marrow suppression, which was effectively managed following symptomatic treatment. He persisted with this treatment protocol for another four cycles, continuing up to 11 November 2024 with a PFS of 8 months. At that point, there was a notable progression of the metastatic bladder tumor, accompanied by an increase in the left pleural effusion (**Figure 1**). Due to continuous hematuria caused by the invasion of the bladder tumor, cystoscopy plus transurethral resection of the lesion was successfully carried out. CRC was confirmed by histopathological analysis of the bladder mass with positivity for CK19, CK20, and CDX-2 (**Figure 2**). Peripheral blood NGS revealed mutations in *RNF43* p.W165*, *TP53* p.A138Gfs*28, and *FBXW7* p.R441W but showed no mutations in *KRAS*, *NRAS*, *BRAF*, *MLH1*, *MSH2*, *MSH2*, or *PMS2*. Given the patient’s compromised postoperative conditions, we opted to employ camrelizumab in combination with capecitabine as the sixth-line therapeutic regimen on 24 November 2024.

## 3 Discussion

A single-arm, phase II prospective trial reported that pemetrexed for CRC patients who have not responded to standard treatments, including 5-fluorouracil, oxaliplatin, and irinotecan, demonstrated an mPFS of 1.6 months and a median overall survival (mOS) of 9.8 months, with an acceptable toxicity profile (9). The REGONIVO study investigated regorafenib combined with a PD-1 inhibitor for MSS/pMMR mCRC patients who failed to respond to chemotherapies, achieving an ORR of 33% and an mPFS of 7.9 months. Notably, all patients who benefited from this regimen were men and had lung metastases, suggesting that these factors could be implications for selecting the potential population for treatment (10). A retrospective study reported that the combination of regorafenib and a PD-1 inhibitor achieved an mPFS of 3 months in MSS CRC patients for third- or later-line treatment (11). In the LEAP-005 study, lenvatinib plus a PD-1 inhibitor for MSS/pMMR mCRC patients yielded an ORR of 22% and an mPFS of 2.3 months in the third-line treatment (12). A real-world study of raltitrexed monotherapy in CRC patients who had previously received fluoropyrimidine-based therapy resulted in an mPFS of 8.5 months and an mOS of 10.2 months (13). In addition, a study found that hyperthermic intraperitoneal chemotherapy with raltitrexed significantly reduced peritoneal metastatic nodes in tumor-bearing mice with CRC (14). In this patient, the PD-1 inhibitor (tislelizumab) combined with regorafenib, pemetrexed, and raltitrexed as fourth-line setting was administered and achieved encouraging results with a PFS of 13 months and tolerable adverse effects.

Chidamide, a novel histone deacetylase (HDAC) inhibitor that targets HDAC1, 2, 3, and 10, demonstrated robust antitumor activity by inducing tumor cell apoptosis and differentiation, inhibiting tumor angiogenesis and metastasis, and enhancing the immune system’s cytotoxic effect on tumors (15). In the CAPability-01 trial, chidamide plus a PD-1 inhibitor with or without anti-VEGF monoclonal antibody showed that mPFS was 3.7 months and mOS was not mature in patients with unresectable chemotherapy-refractory locally advanced or metastatic MSS/pMMR CRC (16). He et al. reported a case of a woman with MSS/pMMR mCRC who received fruquintinib plus a PD-1 inhibitor as third-line treatment and achieved a PFS of 28 months (17). Furthermore, it has been illustrated that a PD-1 inhibitor combined with albumin-bound paclitaxel is effective in metastatic tumors such as upper tract urothelial carcinoma and hypopharyngeal/laryngeal squamous cell carcinoma (18, 19). In light of the aforementioned treatment evidence, we gave this patient fifth-line treatment, including chidamide, fruquintinib, toripalimab, raltitrexed, and nanoparticle albumin-bound paclitaxel (nab-paclitaxel), and he achieved a PFS of 8 months. The side effects were mild and successfully alleviated through symptomatic treatment (**Table 1**).

**Table 1:** Summary of toxicity grades, dose modifications, and supportive care measures in the patient receiving later-line treatment.

Treatments	Adverse events	Toxicity grades	Dose modifications	Supportive care measures
Fourth line	Eczema	Grade 1	None	None
Fifth line	Neutropenia Thrombocytopenia	Grade 3 Grade 2	None	G-CSF

G-CSF, granulocyte colony-stimulating factor.

HDAC inhibitors have emerged as promising agents in enhancing the efficacy of immunotherapy in MSS/pMMR CRC (20, 21). HDAC inhibitors can enhance the expression of major histocompatibility complex class I molecules and related components of the antigen presentation machinery, such as transporter associated with antigen processing and low molecular weight proteins. This enhancement is achieved through inhibition of histone deacetylase activity, which leads to increased histone acetylation and a more open chromatin structure in the promoter regions of these genes (22). In addition, HDAC inhibitors can also modulate the tumor immune microenvironment. They can increase the function of CD8^+^ T cells within the tumor, as well as promote the expression of cytokines such as IFN-γ. This modulation helps reverse tumor cell immune evasion and restore immune surveillance functions (23).

Beyond clinical trials, several case series have highlighted the potential of immunotherapy-based combinations in MSS CRC. Li et al. reported five refractory MSS mCRC cases showing partial response (PR) or SD to fruquintinib plus a PD-1 inhibitor (24). Liu et al. observed that the combination of fruquinitinib with a PD-1 inhibitor yielded PR in six out of eight MSS CRC patients with a PFS ranging from 7 to 21 months (25). In addition, Wang et al. found that regorafenib combined with a PD-1 inhibitor achieved SD in 5 out of 18 MSS CRC patients, with an mPFS of 2 months (26).

This patient subsequently underwent surgery for metastatic bladder cancer, and CRC tissues were confirmed by histopathological test. Given the patient’s weakened postoperative condition, we opted to administer only a PD-1 inhibitor and capecitabine as the sixth-line treatment regimen. The patient is still under follow-up.

## Data Availability

The original contributions presented in the study are included in the article/supplementary material. Further inquiries can be directed to the corresponding author.
